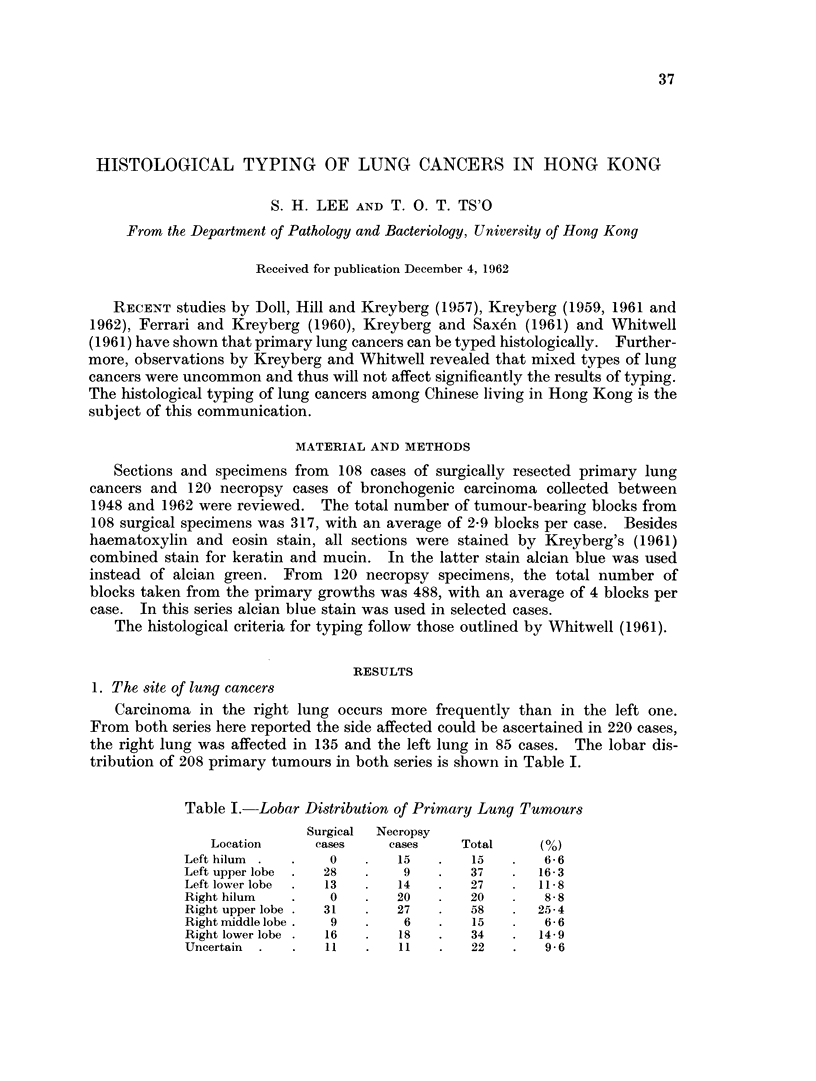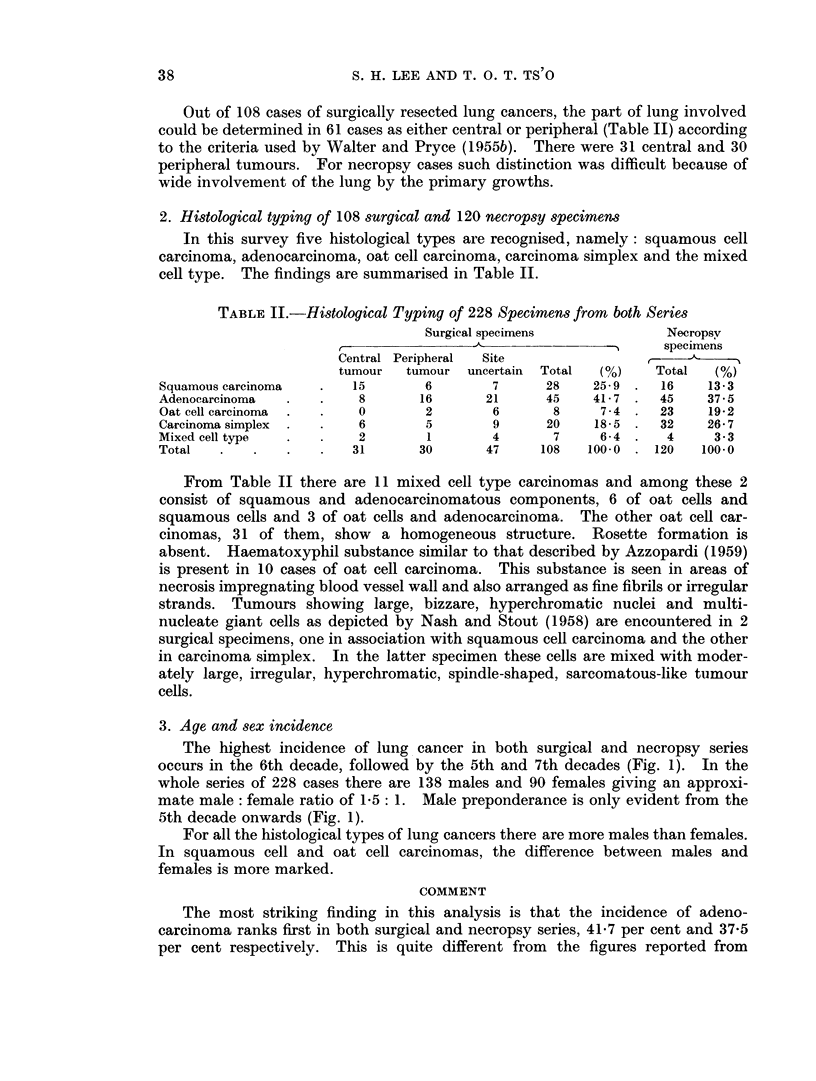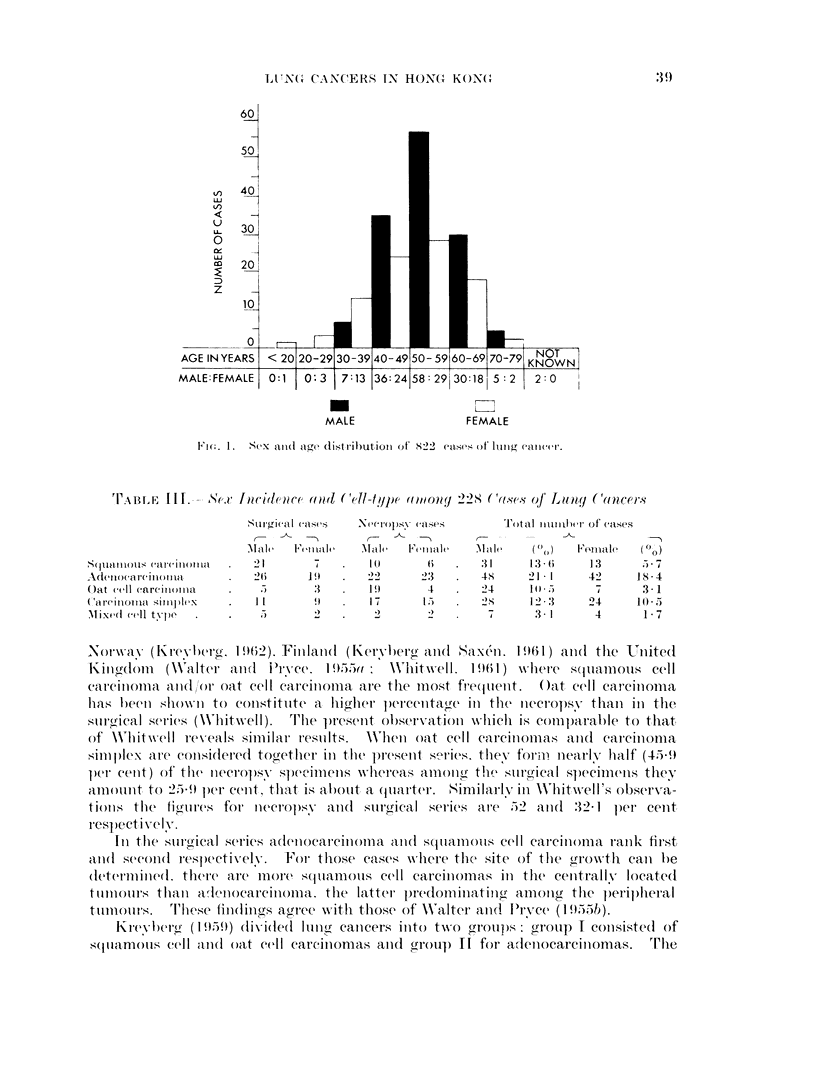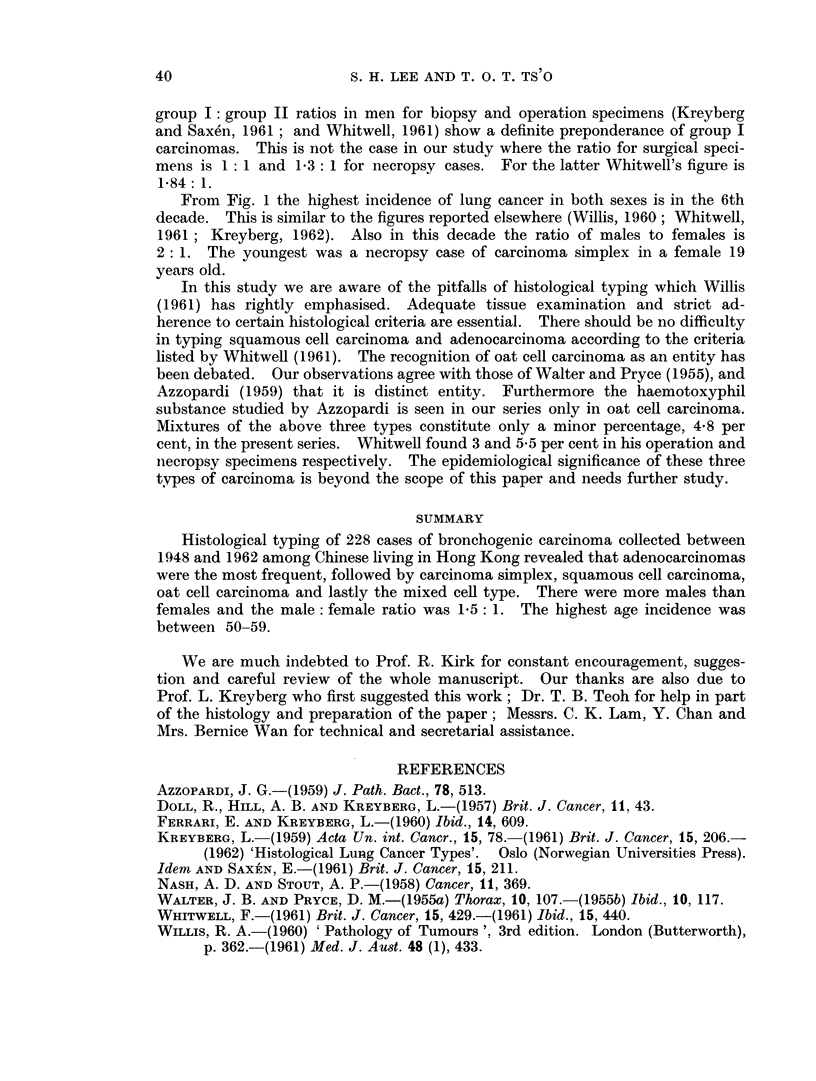# Histological Typing of Lung Cancers in Hong Kong

**DOI:** 10.1038/bjc.1963.4

**Published:** 1963-03

**Authors:** S. H. Lee, T. O. T. Ts'o


### HISTOLOGICAL TYPING OF LUNG CANCERS IN HONG KONG

S. H. LEE AND T. O. T. TS'O

*From the Department of Pathology and Bacteriology, University of Hong Kong*

Received for publication December 4, 1962

RECENT studies by Doll, Hill and Kreyberg (1957), Kreyberg (1959, 1961 and 1962), Ferrari and Kreyberg (1960), Kreyberg and Saxèn (1961) and Whitwell (1961) have shown that primary lung cancers can be typed histologically. Furthermore, observations by Kreyberg and Whitwell revealed that mixed types of lung cancers were uncommon and thus will not affect significantly the results of typing. The histological typing of lung cancers among Chinese living in Hong Kong is the subject of this communication.

### MATERIAL AND METHODS

Sections and specimens from 108 cases of surgically resected primary lung cancers and 120 necropsy cases of bronchogenic carcinoma collected between 1948 and 1962 were reviewed. The total number of tumour-bearing blocks from 108 surgical specimens was 317, with an average of 2·9 blocks per case. Besides haematoxylin and eosin stain, all sections were stained by Kreyberg's (1961) combined stain for keratin and mucin. In the latter stain alcian blue was used instead of alcian green. From 120 necropsy specimens, the total number of blocks taken from the primary growths was 488, with an average of 4 blocks per case. In this series alcian blue stain was used in selected cases.

The histological criteria for typing follow those outlined by Whitwell (1961).

### RESULTS

### 1. *The site of lung cancers*

Carcinoma in the right lung occurs more frequently than in the left one. From both series here reported the side affected could be ascertained in 220 cases, the right lung was affected in 135 and the left lung in 85 cases. The lobar distribution of 208 primary tumours in both series is shown in Table I.

Table I.—*Lobar Distribution of Primary Lung Tumours*

| Location | Surgical cases | Necropsy cases | Total | (%) |
|---|---|---|---|---|
| Left hilum | 0 | 15 | 15 | 6·6 |
| Left upper lobe | 28 | 9 | 37 | 16·3 |
| Left lower lobe | 13 | 14 | 27 | 11·8 |
| Right hilum | 0 | 20 | 20 | 8·8 |
| Right upper lobe | 31 | 27 | 58 | 25·4 |
| Right middle lobe | 9 | 6 | 15 | 6·6 |
| Right lower lobe | 16 | 18 | 34 | 14·9 |
| Uncertain | 11 | 11 | 22 | 9·6 |

Out of 108 cases of surgically resected lung cancers, the part of lung involved could be determined in 61 cases as either central or peripheral (Table II) according to the criteria used by Walter and Pryce (1955*b*). There were 31 central and 30 peripheral tumours. For necropsy cases such distinction was difficult because of wide involvement of the lung by the primary growths.

2. *Histological typing of* 108 *surgical and* 120 *necropsy specimens*

In this survey five histological types are recognised, namely : squamous cell carcinoma, adenocarcinoma, oat cell carcinoma, carcinoma simplex and the mixed cell type. The findings are summarised in Table II.

TABLE II.—*Histological Typing of* 228 *Specimens from both Series*

| | Surgical specimens | | | | | Necropsy specimens | |
|---|---|---|---|---|---|---|---|
| | Central tumour | Peripheral tumour | Site uncertain | Total | (%) | Total | (%) |
| Squamous carcinoma | 15 | 6 | 7 | 28 | 25·9 | 16 | 13·3 |
| Adenocarcinoma | 8 | 16 | 21 | 45 | 41·7 | 45 | 37·5 |
| Oat cell carcinoma | 0 | 2 | 6 | 8 | 7·4 | 23 | 19·2 |
| Carcinoma simplex | 6 | 5 | 9 | 20 | 18·5 | 32 | 26·7 |
| Mixed cell type | 2 | 1 | 4 | 7 | 6·4 | 4 | 3·3 |
| Total | 31 | 30 | 47 | 108 | 100·0 | 120 | 100·0 |

From Table II there are 11 mixed cell type carcinomas and among these 2 consist of squamous and adenocarcinomatous components, 6 of oat cells and squamous cells and 3 of oat cells and adenocarcinoma. The other oat cell carcinomas, 31 of them, show a homogeneous structure. Rosette formation is absent. Haematoxyphil substance similar to that described by Azzopardi (1959) is present in 10 cases of oat cell carcinoma. This substance is seen in areas of necrosis impregnating blood vessel wall and also arranged as fine fibrils or irregular strands. Tumours showing large, bizzare, hyperchromatic nuclei and multinucleate giant cells as depicted by Nash and Stout (1958) are encountered in 2 surgical specimens, one in association with squamous cell carcinoma and the other in carcinoma simplex. In the latter specimen these cells are mixed with moderately large, irregular, hyperchromatic, spindle-shaped, sarcomatous-like tumour cells.

3. *Age and sex incidence*

The highest incidence of lung cancer in both surgical and necropsy series occurs in the 6th decade, followed by the 5th and 7th decades (Fig. 1). In the whole series of 228 cases there are 138 males and 90 females giving an approximate male : female ratio of 1·5 : 1. Male preponderance is only evident from the 5th decade onwards (Fig. 1).

For all the histological types of lung cancers there are more males than females. In squamous cell and oat cell carcinomas, the difference between males and females is more marked.

### COMMENT

The most striking finding in this analysis is that the incidence of adenocarcinoma ranks first in both surgical and necropsy series, 41·7 per cent and 37·5 per cent respectively. This is quite different from the figures reported from

FIG. 1. Sex and age distribution of 822 cases of lung cancer.

TABLE III.—*Sex Incidence and Cell-type among 228 Cases of Lung Cancers*

| | Surgical cases | | Necropsy cases | | Total number of cases | | | |
|---|---|---|---|---|---|---|---|---|
| | Male | Female | Male | Female | Male | (%) | Female | (%) |
| Squamous carcinoma | 21 | 7 | 10 | 6 | 31 | 13·6 | 13 | 5·7 |
| Adenocarcinoma | 26 | 19 | 22 | 23 | 48 | 21·1 | 42 | 18·4 |
| Oat cell carcinoma | 5 | 3 | 19 | 4 | 24 | 10·5 | 7 | 3·1 |
| Carcinoma simplex | 11 | 9 | 17 | 15 | 28 | 12·3 | 24 | 10·5 |
| Mixed cell type | 5 | 2 | 2 | 2 | 7 | 3·1 | 4 | 1·7 |

Norway (Kreyberg, 1962), Finland (Kerybeg and Saxén, 1961) and the United Kingdom (Walter and Pryce, 1955*a*; Whitwell, 1961) where squamous cell carcinoma and/or oat cell carcinoma are the most frequent. Oat cell carcinoma has been shown to constitute a higher percentage in the necropsy than in the surgical series (Whitwell). The present observation which is comparable to that of Whitwell reveals similar results. When oat cell carcinomas and carcinoma simplex are considered together in the present series, they form nearly half (45·9 per cent) of the necropsy specimens whereas among the surgical specimens they amount to 25·9 per cent, that is about a quarter. Similarly in Whitwell's observations the figures for necropsy and surgical series are 52 and 32·1 per cent respectively.

In the surgical series adenocarcinoma and squamous cell carcinoma rank first and second respectively. For those cases where the site of the growth can be determined, there are more squamous cell carcinomas in the centrally located tumours than adenocarcinoma, the latter predominating among the peripheral tumours. These findings agree with those of Walter and Pryce (1955*b*).

Kreyberg (1959) divided lung cancers into two groups; group I consisted of squamous cell and oat cell carcinomas and group II for adenocarcinomas. The group I : group II ratios in men for biopsy and operation specimens (Kreyberg and Saxén, 1961 ; and Whitwell, 1961) show a definite preponderance of group I carcinomas. This is not the case in our study where the ratio for surgical specimens is 1 : 1 and 1·3 : 1 for necropsy cases. For the latter Whitwell's figure is 1·84 : 1.

From Fig. 1 the highest incidence of lung cancer in both sexes is in the 6th decade. This is similar to the figures reported elsewhere (Willis, 1960 ; Whitwell, 1961 ; Kreyberg, 1962). Also in this decade the ratio of males to females is 2 : 1. The youngest was a necropsy case of carcinoma simplex in a female 19 years old.

In this study we are aware of the pitfalls of histological typing which Willis (1961) has rightly emphasised. Adequate tissue examination and strict adherence to certain histological criteria are essential. There should be no difficulty in typing squamous cell carcinoma and adenocarcinoma according to the criteria listed by Whitwell (1961). The recognition of oat cell carcinoma as an entity has been debated. Our observations agree with those of Walter and Pryce (1955), and Azzopardi (1959) that it is distinct entity. Furthermore the haemotoxyphil substance studied by Azzopardi is seen in our series only in oat cell carcinoma. Mixtures of the above three types constitute only a minor percentage, 4·8 per cent, in the present series. Whitwell found 3 and 5·5 per cent in his operation and necropsy specimens respectively. The epidemiological significance of these three types of carcinoma is beyond the scope of this paper and needs further study.

### SUMMARY

Histological typing of 228 cases of bronchogenic carcinoma collected between 1948 and 1962 among Chinese living in Hong Kong revealed that adenocarcinomas were the most frequent, followed by carcinoma simplex, squamous cell carcinoma, oat cell carcinoma and lastly the mixed cell type. There were more males than females and the male : female ratio was 1·5 : 1. The highest age incidence was between 50–59.

We are much indebted to Prof. R. Kirk for constant encouragement, suggestion and careful review of the whole manuscript. Our thanks are also due to Prof. L. Kreyberg who first suggested this work ; Dr. T. B. Teoh for help in part of the histology and preparation of the paper ; Messrs. C. K. Lam, Y. Chan and Mrs. Bernice Wan for technical and secretarial assistance.

### REFERENCES

Azzopardi, J. G.—(1959) *J. Path. Bact.*, **78**, 513.
Doll, R., Hill, A. B. and Kreyberg, L.—(1957) *Brit. J. Cancer*, **11**, 43.
Ferrari, E. and Kreyberg, L.—(1960) *Ibid.*, **14**, 609.
Kreyberg, L.—(1959) *Acta Un. int. Cancr.*, **15**, 78.—(1961) *Brit. J. Cancer*, **15**, 206.—(1962) 'Histological Lung Cancer Types'. Oslo (Norwegian Universities Press).
*Idem* and Saxén, E.—(1961) *Brit. J. Cancer*, **15**, 211.
Nash, A. D. and Stout, A. P.—(1958) *Cancer*, **11**, 369.
Walter, J. B. and Pryce, D. M.—(1955*a*) *Thorax*, **10**, 107.—(1955*b*) *Ibid.*, **10**, 117.
Whitwell, F.—(1961) *Brit. J. Cancer*, **15**, 429.—(1961) *Ibid.*, **15**, 440.
Willis, R. A.—(1960) 'Pathology of Tumours', 3rd edition. London (Butterworth), p. 362.—(1961) *Med. J. Aust.* **48** (1), 433.